# Report on the Meteorological Characteristics, and More Important Diseases Prevalent in Chicago, during the Summer of 1856

**Published:** 1856-11

**Authors:** N. S. Davis


ARTICLE III.—*Report on the Meteorological Characteristics, and More Important Diseases Prevalent in Chicago, during the Summer of* 1856; *presented to the Cook County Medical Society, Oct.* 7*th*, 1856. By N. S. DAVIS, M.D.

It is now four years since I commenced a careful series of observations with a view of determining what connection, if any, there was between the atmospheric or meteorological conditions of each season, and the prevalence of different forms of disease. The results of these observations have been read to this Society from time to time, and may be found recorded in the *North-Western Medical Journal*, Volumes II. III. and IV. The pressure of other duties, during the past summer, has prevented me from keeping records so much in detail as in some previous seasons. Still, they are sufficient for my present purpose.

The chief meteorological characteristics of the past six months, ending October 1st, may be stated in general terms as follows, viz.:—

*First*—The mean temperature of the whole season has been below the average of a series of years, so much so, as to be called emphatically a "Cold Summer."

*Second*—The atmospheric moisture has been fully equal to the ordinary average for this locality, being much above that of 1853, and slightly below that of 1854.

*Third*—The amount of rain and the number of rainy days, have varied but little from the average of a series of years.

Leaving these general statements, which, as I have stated in previous reports, are of little etiological value, we may find, in the detail of facts, such as have an interesting and important bearing on the prevalence and specific character of disease. The season furnished no warm days worthy of note until the 19th of June. From the 19th to the 22d, the temperature was high, the atmosphere above the average of moisture, and the wind nearly all the time from the South and South-West, that is, from the prairie in this locality. The high temperature, coupled with moisture and South winds, gave to the atmosphere those qualities which we denominate *sultry* or oppressive. On the evening of the 22d, there were showers of rain, with changeable winds and a lower temperature. The atmosphere, however, remained damp and oppressive during all the remaining days of June. The month of July, though furnishing a low average temperature, was, nevertheless, characterized by peculiar atmospheric phenomena. Moderate rains occurred almost every week, each one immediately followed by a higher temperature, greater dampness, and generally South or South-West wind. After one or two days of this sultry character, the atmosphere, generally, became cooler and dryer until the next showers or rainy days. The month of August was unusually cold and dry. Showers occurred but three or four times during the month; but, as in July, each was uniformly followed by a day of higher temperature, greater moisture, and, consequently, a more oppressive or relaxing atmosphere. After these, one or two days it became cooler, and continued so until the next fall of rain. There were several days in August cool enough to make an overcoat comfortable. The first week in September was recorded as "warm, wet, and oppressive." The remainder of the month was unusually cold, but still each rainy day was followed by one or two during which the atmosphere was warmer and excessively damp.

In glancing back over the past four years, I find each presenting atmospheric peculiarities which strikingly distinguish it from all the others. Thus, the summer of 1853 was distinguished by two periods of excessive heat; one in June, and the other in August; but both accompanied by a *dry* and bracing atmosphere, particularly throughout the North-Western States. The temperature of July was a little below the average, but the atmosphere remained remarkably dry and pleasant, in this section of the country, throughout the whole summer. It was also a season of unparalleled good health. The ratio of deaths in this city, during that year, was only one in fifty of the population.

The next summer (1854) was characterized by excessive and protracted heat, coupled with unusual dampness of the atmosphere, amounting often almost to saturation; yet rains were infrequent, and in many parts of this and the neighboring States the ground became so dry, during the latter part of summer and autumn, as to destroy vegetation. It was a season of unusual sickness throughout the country. This city was scourged by a severe epidemic of cholera, which commenced as early as the last of April, and swelled the aggregate mortality of that year to one in nineteen of the population.

The summer of 1855, was, in many respects, the reverse of that of 1854. The mean temperature was below the average for a series of years. There was at no time during the season more than four consecutive days of high temperature. The order of atmospheric phenomena was, generally, a gradually-increasing temperature for three or four successive days, then showers, with a sudden and extreme change to cold, causing the mercury in the thermometer sometimes to fall 20° in a few hours. The low temperature generally continued from one to two days. The atmospheric moisture was, most of the time, above the average. The season, as a whole, was cold and damp, the rains being frequent and almost invariably accompanied or immediately followed by a change to a much lower atmospheric temperature. The summer of the present year (1856) has been much like that of 1855, with this important exception, namely, the rains have been almost uniformly accompanied or immediately followed by one or two days of increased, instead of diminished, atmospheric temperature. The winters of 1854–'5, and 1855–'6, were both unusually cold, and accompanied by more snow than during many years previous. The snow of the latter winter, particularly, was not only unusual in quantity, but it remained on the ground, accompanied by cold weather, until a very late period of the spring. The summer and autumn of 1855 was distinguished by a greater prevalence of periodical fevers, throughout this and the adjoining States, than had occurred during many years previous. In this city there were many cases of dysentery between the middle of July and the middle of September. But, as a whole, the year was one of good health in the city, the ratio of deaths being a fraction less than one in forty of the population.

During the present season, the health of the city has continued unusually good, until the warm, sultry days I have described between the 18th and 22d of June. At that time, I noticed a sudden and marked increase in the number of attacks of diarrhœa and cholera morbus. This increase was more marked among children under two years of age than any other class. I noticed, at the same time, in many of the cases, decided symptoms of cerebral disease complicating the intestinal disorder. In the Catholic Orphan Asylum, eight children were attacked during the 20th and 21st of June with active vomiting and purging. Two of them were accompanied by heat in the head, contraction of the pupils, and severe convulsions. Both died within 48 hours, but the other cases recovered. From the 25th of June to the 15th of July, diarrhœa and cholera morbus continued to prevail, chiefly among young children; and I noticed several cases complicated with lobular pneumonia, thereby adding much to the danger of the patient. During the last half of July, the attacks of diarrhœa and dysentery, among children under two years of age, were so much increased as to almost merit the title of an epidemic. The adult portion of the population, however, remained as much exempt from sickness as in the most healthy seasons. During the month of August new attacks were less frequent among children, but many of those who sickened in July, especially among the more ignorant classes, being neglected or badly treated, the disease assumed a more chronic form and terminated fatally during the month of August, thereby increasing the ratio of mortality for that month much beyond the ratio of new attacks. During the month of September, the cases of active diarrhœa and cholera morbus, among children, nearly ceased to recur. Dysentery, also, prevailed to a moderate extent, both among children and adults, during all the months of July, August, and September. The period of its most active prevalence, was during the last half of July. From the 19th to the 31st of that month, there occurred within the circle of my own practice 38 cases of dysentery, and 34 of diarrhœa and cholera morbus. In the latter number, is included a case which presented every symptom and feature of spasmodic cholera. The patient was a printer, aged about 35 years. He was attacked with vomiting, purging of serum or rice-water, severe muscular cramps, and all the symptoms belonging to the most active cholera, about the middle of the night of the 27th July.

When I saw him at 10 o'clock A.M. his skin was cool and corrugated; his eyes sunken, and features contracted; his voice husky and feeble; his pulse quick and very small; his discharges frequent, still serous, with almost constant distressing cramps in his extremities. During the months of July and August, I saw three other similar cases. None of them proved fatal; but, had it been a season, when epidemic cholera was prevailing, there would have been no hesitation in classing them under that head. Other practitioners in the city have informed me of five or six cases coming under their observation, with symptoms equally well marked, and two of which died in collapse. The extent to which acute bowel affections influenced the aggregate mortality during the past summer will be strikingly exhibited by the following statistics:—

| | Whole No. of Deaths in each Month. | No. from Bowel Affections |
|---|---|---|
| January, | 111 | 11 |
| February, | 104 | 11 |
| March, | 92 | 15 |
| April, | 108 | 18 |
| May, | 128 | 20 |
| June, | 142 | 40 |
| July, | 425 | 310 |
| August, | 342 | 234 |
| September, | 224 | 68 |

At least four-fifths of the excess of mortality in July and August, over May and June, took place in children under three years of age, and was occasioned by cholera infantum and acute dysentery. This high ratio of mortality among children occurred the present season not in this city alone; but, if I mistake not, the same has occurred, in a greater or less degree, in nearly all the cities of our country. Notwithstanding the great number of deaths among young children from bowel affections, during the months of July and August, I have failed to notice anything peculiar in the symptoms or progress of these affections as they have come under my observation. Except those cases occurring during the last week in June and the first few days of July, which were plainly complicated with cerebral or pulmonary inflammation, the attacks, whether in the form of cholera morbus, diarrhœa, or dysentery, were not unusually severe, and almost always yielded readily to appropriate treatment, if adopted in the early stage of the disease. But my own daily observations have satisfied me, that full two-thirds of the infantile mortality in this city has been occasioned by simple neglect or positive bad management on the part of mothers and nurses. Of 45 children brought to my office, for advice during the month of August, affected with diarrhœa or dysentery, 34 had been laboring under the disease from two to six weeks, and had become extremely emaciated and anemic, with symptoms of ulceration of the mucous membranes, and, in many, enlargement of the mesenteric glands. When asked why they had not applied for advice sooner, the reply was pretty uniform, "That the child was *teething*, and they did not think it good to stop the discharges too soon." Sometimes they had positively added to the mischief, by giving every three or four days a dose of castor oil, and in some cases, also, repeated doses of vermifuge medicines. In the City Sexton's Report for the month of August, no less than 54 are recorded as having died "from *teething*." It is comparatively rare to find a child affected with disease of any kind between the ages of four and eighteen months, that the mother or nurse does not attribute to the influence of the first dentition. And the idea that such cases, especially when they present the form of diarrhœa or cholera morbus, should not be promptly arrested, occasions an annual sacrifice of more than 100 children in this city alone.

Very few cases, of either diarrhœa or cholera morbus, came under my observation in the early stage of the disease which were not promptly relieved by small doses of calomel combined with opiates, and given at intervals of two, four, or six hours, until from three to six doses had been taken. When the vomiting was frequent and active, a solution of bi-carb. soda, ʒss. with acetas morph. 1gr. in water ℥ij. given in doses of from 10 to 20 drops immediately after each turn of vomiting, has aided much to allay the gastric irritation. These means generally arrested the vomiting and purging within twenty-four hours, and then were discontinued. If the opiates check the action of the kidneys as well as the bowels, as is frequently the case, the child may continue unusually sleepy, with nervous startings, for from twelve to twenty-four hours after they have been discontinued. These unpleasant symptoms are generally removed in a few hours by applying warm fomentations over the lower part of the abdomen, and giving internally from 5 to 10 drops of nitrous ether (spts. nit. dulc.) in a spoonful of strong infusion of common tea. In from twelve to twenty-four hours after the discharges have been checked, and the exhibiion of remedies for that purpose suspended, the evacuations from the bowels again recur, but are generally less copious and watery, of a dark green or yellow color, and not so frequent as at first. I now inquire carefully concerning the nature of the discharges, and the general condition of the patient. If I find the matter discharged to be very thin, still copious in quantity, and without the intermixture of mucous, I often prescribe the following, viz.:—

℞—Acetas Plumbi, . . ℈j.

Acetas Morph. . . 1 gr.

Acetic Acid, . . . ʒj.

Water, . . . ℥ij.

Mix.

Dose from 10 to 20 drops, to a child from six to eighteen months old, repeated every 4 or 6 hours, according to the frequency of the evacuations. If the patient is feverish, indicated by heat of skin and quickness of pulse, I often give, in addition to the solution, a powder night and morning, containing half a grain of calomel and one-third of a grain of pulv. Doveri. In some instances, during the past summer, I have found the fever, following an attack of diarrhœa or cholera morbus, to present a distinctly remittent tendency. In all such instances I have added from one-third of a grain to one grain of tanate of quinine to each dose of the powders just mentioned, and with the happiest results. In other cases, where the first onset of the disease has caused greater prostration, indicated by a continued cool skin, sunken eyes, feeble pulse, scanty urine, and yet too frequent serous discharges, instead of giving the solution of acetas plumbi, as before mentioned, I have directed the following, viz.:—

℞—Erigiron Canadensis (flea-bane), ℥ss.
Tanate of Quinine, . . . . 20grs.
Sulph. Morphine, . . . . 1gr.

Mix, and add boiling water 1 pint. Stir it up well, and let it stand until cold, then give from 1 to 2 tea-spoonsful of this infusion every 2, 3, or 4 hours, according to the age of the patient. A score of cases might be detailed in which this seemed to produce more benefit than any other combination of remedies I could devise. In three cases, where I found the little patients, after a severe attack of cholera morbus, extremely depressed, the mind torpid and drowsy, the face pale and contracted, the almost entire suppression of urine, and still a continuance of frequent watery evacuations of a greenish color, the above infusion was given every two hours, with 10 drops of nitrous ether added to each dose, and with a more rapid and permanent improvement than I have obtained from other remedies under the same circumstances.

In many of the attacks met with during the past summer, after the first active vomiting and purging had ceased, the discharges from the bowels would continue frequent, but small in quantity, more or less mixed with mucus, and sometimes streaked with blood, and the expulsion accompanied by straining or tenesmus. In the great majority of such cases, the following emulsion was found most beneficial, viz.:—

| | | |
|---|---|---|
| ℞—Ol. Terebinth, . . | | ʒj. |
| Tinct. Opii, . . . | | ʒj. |
| Pulv. G. Arabac, | aa | ʒiij. |
| White Sugar, | | |
| Rub together, and add | | |
| Mint Water, . . . | | ℥ij. |

Of this, from 10 drops to half a tea-spoonful may be given at a dose, according to the age of the patient, and repeated every 2, 4, or 6 hours, until the discharges become normal in frequency and consistence. In several of the cases met with during the month of July and the early part of August, a distinctly remitting type of fever existed in connection with the enteric irritation. Its symptoms were not always prominently developed, but frequently consisted in simple increased heat of the skin, a more frequent pulse, and more thirst during the afternoon and evening. In such cases, the exhibition of from half a grain to two grains of tanate of quinine, at intervals of three hours during the morning of each day, in addition to the other remedies, generally aided much in producing a rapid and complete recovery.

As has been previously stated, very many of the cases of diarrhœa and cholera morbus that came under my care, had already assumed a chronic form, accompanied by great emaciation, a feeble pulse, and sometimes cold extremities. The alvine evacuations varied much in different cases. In some they were copious, thin as water, and varying in color from dark green to a whitish or pale slate. In some there was mixed with the thin evacuations, white specks or flockuli, and, in other cases, they were small in quantity, frequently repeated, and composed chiefly of mucous or muco-purulent matter, and occasionally streaked with blood. In some, the abdomen was full, tense, and tympanitic, while in others it was flaccid and empty. Of course the remedial agents employed were various. In those cases accompanied by watery evacuations, cool extremities, flaccid abdomen, and either no fever at all or its recurrence only in paroxysms, the infusion of Erigiron Canadensis with quinine, and a minute quantity of morphine, was generally found very useful. In addition, I often gave each night and morning a powder, composed of from one-quarter to one-half of a grain of acetate of lead, and the eighth of a grain of opium. If the intestinal discharges were light colored, the addition of half a grain of calomel or mercury with chalk, to the powder given at night, generally soon effected a favorable change. I met with several cases like those last described, in which the capillary circulation was very feeble, giving the skin a livid or purple color, with unusual drowsiness and slow respiration. In such the circulation and respiration were too feeble to permit the proper interchange of carbonic acid gas and oxygen in the lungs, which added much to the dangerous tendency of the original disease.

Knowing that chloride of sodium (common salt) possessed the power to increase the capacity of the blood for absorbing oxygen, and promoted the capillary circulation, I prescribed the following mixture in several cases with marked advantage, viz.:—

| | |
|---|---|
| ℞—Chloride Sodium, . . | ʒij. |
| Tinct. Opii, . . . . | ʒj. |
| Sulph. Quinine, . | 10grs. |
| Mucilage of G. Arabac, | ℥iij. |
| Mix. | |

Dose, from 20 to 30 drops, every 2, 3, or 4 hours, according to the age of the child and the frequency of the discharges.

In most of the cases accompanied by a tumid or tympanitic abdomen, with frequent discharges, consisting in part of mucus, the emulsion of oil of turpentine and laudanum seldom failed to afford relief.

In three very protracted cases, in which I supposed the diarrhœa to be complicated with more or less enlargement of the mesenteric glands, the following was given and continued for two or three weeks, with the happiest effects, viz. :—

℞—Glycerine, . . . ℥ij.

Syrup of Iodide of Iron, ʒij.

Sulph. Morphine, . . 1gr.

Mix,

And give from 10 to 30 drops, every 4 or 6 hours, in a teaspoonful of sweetened water.

To illustrate further the treatment of this important class of diseases, would extend this report to an undue length. It will be observed that we restrict the use of mercurials mostly to the first stage, and then give but a limited number of small doses.

The pathological views of Dr. James Stewart, in his recent Prize Essay on the subject of Cholera Infantum, are directly calculated to encourage an excessive use of this potent class of remedies. Regarding a high atmospheric temperature with excessive moisture, as the efficient agents in determining the development of the disease, Dr. Stewart supposes them to diminish the interchange of carbon and oxygen in the lungs and thereby cause an excessive action of the liver, as the next great organ for eliminating effete carbonaceous matter. This excessive action leads to undue engorgement of the hepatic structure, and subsequently to engorgement of the whole portal system of vessels. Hence, he makes congestion or engorgement of the liver, the first link in the chain of morbid action which results in cholera infantum. I have no confidence whatever in this theory. There is no doubt, but a high atmospheric temperature and moisture constitute a part of the circumstances necessary for the development of this disease; but it is equally necessary that the preceding winter should have been sufficiently cold and protracted to make a wide contrast between the mean temperature of winter and that of summer. And it is much more probable that the heat of summer, following a cold and protracted winter, induces intestinal fluxes of all kinds, more by inducing conjointly undue relaxation of all the textures of the body, with excessive susceptibility, and excessive elimination of saline elements of the blood through the skin, than by any mere local action, or want of action in the liver. It is true, that we meet with cases of infantile diarrhœa, with so much inactivity of the liver that the evacuations show no evidence of the presence of bile; and we meet with many other cases in which the flow of bile seems greatly increased: but neither the constant symptoms during life, nor the post mortem appearances, afford any evidence that the liver is either primarily or prominently affected in this disease.

It will be seen that quinine has constituted an ingredient in many of my prescriptions for the diarrhœa of children during the past summer.

Two or three years since, Dr. DeLaskie Miller called the attention of this Society to the beneficial effects of this agent in the treatment of the summer complaints of children. And very recently Dr. Wright, Professor of Physiology and Pathology in the Memphis Medical College, has published an article in the *Memphis Medical Recorder*, not only advocating strongly the beneficial effects of quinine in the cholera infantum and diarrhœa of children, but claiming that these affections are only disguised remittents, or, in other words, the results of the action of malaria on the systems of young children.

My own observations do not permit me to endorse the extreme views of Dr. Wright, although I have several times seen well-marked remittent fever in connection with cholera morbus and diarrhœa in children, and have seen the cases promptly cured by quinine and opium. But, independent of all true periodicity, there is, in a large proportion of the cases of infantile diarrhœa, that peculiar relaxation or loss of tone in all the structures of the body, coupled with increased susceptibility or mobility, which makes the sedative and tonic qualities of quinine particularly applicable in their treatment: and hence it is that I have so often coupled it with other ingredients designed especially to counteract the local intestinal irritation and discharges.

The remainder of the report, relating to the prevalence of fevers and dysentery, must be deferred until the next number of the *Journal.*

